# Case Report: Novel AK2 variant causing reticular dysgenesis with hemophagocytic lymphohistiocytosis-like syndrome and invasive aspergillosis

**DOI:** 10.3389/fimmu.2026.1787177

**Published:** 2026-04-10

**Authors:** Anas A. Hadid, Lana A. Shaiba, Adnan Hadid, Alkhansaa M. Alhag, Odai Alyateem, Nora Al-Saud, Badr Sobaih

**Affiliations:** 1 College of Medicine, Dar Al Uloom University, Riyadh, Saudi Arabia; 2 Department of Pediatrics, College of Medicine, King Saud University, Riyadh, Saudi Arabia; 3 Department of Neonatology, King Saud University Medical City, Riyadh, Saudi Arabia

**Keywords:** adenylate kinase 2, case report, hemophagocytic lymphohistiocytosis, invasive aspergillosis, reticular dysgenesis, severe combined immunodeficiency

## Abstract

**Background:**

Reticular dysgenesis (RD) is the most severe form of severe combined immunodeficiency, caused by biallelic AK2 mutations. The association between RD and hemophagocytic lymphohistiocytosis (HLH) remains poorly characterized, with only one prior case reported.

**Case presentation:**

A female neonate born to consanguineous parents presented on day 5 of life with fever and profound pancytopenia. Whole-exome sequencing identified a novel homozygous AK2 missense variant (c.79G>C; p.Gly27Arg). The clinical course was complicated by G-CSF-refractory neutropenia, recurrent sepsis, fulminant Aspergillus flavus sinusitis with craniofacial destruction, and HLH-like syndrome (hyperferritinemia 2,258 ng/mL, elevated sCD25 8,450 U/mL). Despite an available HLA-matched sibling donor, hematopoietic stem cell transplantation was precluded by active infection. The patient died at 5 months of age.

**Conclusion:**

This is the first report of neonatal RD with concurrent HLH-like syndrome and invasive fungal infection. The novel p.Gly27Arg variant expands the AK2 mutation spectrum. These findings suggest immune dysregulation in RD extends beyond immunodeficiency to include inflammatory dysregulation.

## 1 Introduction

Reticular dysgenesis (RD; OMIM #267500) is the most severe form of severe combined immunodeficiency (SCID), accounting for approximately 2% of SCID cases (1, 2). RD results from biallelic loss-of-function mutations in the adenylate kinase 2 (AK2) gene on chromosome 1p35.1, which encodes an enzyme critical for mitochondrial energy metabolism and hematopoietic cell differentiation (3–5). The classic phenotype includes profound agranulocytosis, severe T- and B-cell lymphopenia, and bilateral sensorineural deafness (1, 2). Without hematopoietic stem cell transplantation (HSCT), RD is universally fatal (6, 7).

Hemophagocytic lymphohistiocytosis (HLH) is characterized by excessive immune activation with uncontrolled cytokine release (8). Primary HLH encompasses not only classical familial HLH due to lymphocyte cytotoxic defects (e.g., PRF1, UNC13D, STX11, STXBP2 mutations), but also HLH caused by other primary genetic etiologies including inflammasome defects, metabolic disorders, and inborn errors of immunity (9, 10). While primary immunodeficiencies can trigger HLH through multiple mechanisms (9), the association with RD has been reported only once a 2.5-month-old infant with hemophagocytosis and unusual histiocyte-like cells (11). That case lacked invasive fungal infection.

Here, we present a neonate with RD caused by a novel AK2 missense variant (p.Gly27Arg), whose clinical course was complicated by both HLH-like syndrome and fulminant invasive aspergillosis. This combination has not been previously described and expands both the molecular and clinical spectrum of AK2 deficiency.

## 2 Case description

### 2.1 Patient information and presentation

A female infant was born at 37 weeks’ gestation via cesarean section to first-degree consanguineous Saudi parents, with a birth weight of 2.2 kg. The antenatal period was unremarkable. She was discharged on day 4 but presented to our neonatal intensive care unit on day 5 with fever (38.5 °C), lethargy, poor feeding, and weak cry. Family history was negative for immunodeficiency or early childhood deaths.

### 2.2 Clinical findings

Physical examination revealed a lethargic neonate with fever, mild hepatomegaly (2 cm below costal margin), and no lymphadenopathy or rash. Initial investigations demonstrated profound pancytopenia: white blood cell count 0.8 × 10^9^/L, absolute neutrophil count <100 cells/μL, absolute lymphocyte count 0.3 × 10^9^/L, hemoglobin 7.8 g/dL, and platelets 45 × 10^9^/L. Blood cultures grew Klebsiella pneumoniae.

Lymphocyte subset analysis by flow cytometry was not feasible due to profound lymphopenia; immunological assessment was therefore based on complete blood count with differential, serum immunoglobulin levels, and bone marrow evaluation. Serum immunoglobulins showed low IgM (0.12 g/L; reference: 0.2–1.0) with preserved IgG (6.8 g/L, likely maternal). Bone marrow aspiration revealed marked hypocellularity (<10%) with absent myeloid precursors, prominent histiocytic infiltration, atypical histiocyte-like cells, and hemophagocytic activity. No malignancy was detected.

### 2.3 Timeline

**Table 1** presents the chronological timeline of key clinical events from birth through outcome.

**Table 1:** Timeline of clinical events.

Time point	Clinical events and interventions
Day 0	Birth at 37 weeks via cesarean; birth weight 2.2 kg; unremarkable early neonatal course
Day 5	Presentation with fever, lethargy; pancytopenia identified; Klebsiella pneumoniae sepsis; initiated meropenem, vancomycin, G-CSF
Day 7	CBC confirms severe pan-lymphopenia; bone marrow shows hypocellularity with hemophagocytic activity; WES ordered
Day 14	Respiratory failure requiring mechanical ventilation; recurrent sepsis (Pseudomonas aeruginosa)
Day 18	Palatal ulceration noted; Aspergillus flavus cultured; voriconazole and caspofungin initiated
Day 21	WES confirms novel homozygous AK2 variant c.79G>C (p.Gly27Arg); parental carrier status confirmed
Day 28	HLH-like syndrome diagnosed: ferritin 2,258 ng/mL; dexamethasone initiated
Day 33	Dexamethasone discontinued due to worsening fungal infection; CT shows extensive craniofacial destruction
Day 45	HLA-matched sibling donor identified; HSCT deemed unfeasible due to active infection and instability
5 months	Transition to comfort care; death

## 3 Diagnostic assessment

### 3.1 Genetic analysis

Given severe agranulocytosis, pan-lymphopenia, and consanguinity, whole-exome sequencing was performed. This identified a novel homozygous AK2 missense variant: c.79G>C (p.Gly27Arg) in exon 2 (NM_001625.4). Sanger sequencing confirmed parental heterozygous carrier status.

In silico analysis (SIFT, PolyPhen-2, CADD, REVEL) uniformly predicted this variant as deleterious. The glycine at position 27 is located within the highly conserved Walker A motif (P-loop) of the nucleotide-binding domain, showing complete conservation across vertebrates. The variant is absent from gnomAD (v4.0), ClinVar, and HGMD databases.

### 3.2 HLH-like syndrome assessment

The patient met 5 of 8 HLH-2004 diagnostic criteria (8): persistent fever >38.5 °C, progressive cytopenias, hyperferritinemia (peak 2,258 ng/mL; reference <200), hypertriglyceridemia (4.8 mmol/L; reference <1.7), and elevated soluble IL-2 receptor (8,450 U/mL; reference <2,400). Additional inflammatory markers were elevated: CRP 160 mg/L, procalcitonin 54.27 ng/mL.

## 4 Therapeutic intervention

Initial management included broad-spectrum antibiotics (meropenem, vancomycin), G-CSF (escalating to 10 μg/kg/day without neutrophil recovery), IVIG (400 mg/kg every 3 weeks), and Pneumocystis jirovecii prophylaxis. Recurrent sepsis (Pseudomonas aeruginosa, Staphylococcus epidermidis) required multiple antibiotic regimen changes.

For invasive aspergillosis, combination antifungal therapy was initiated: voriconazole (9 mg/kg twice daily loading, then 8 mg/kg twice daily) and caspofungin (70 mg/m^2^ loading, then 50 mg/m^2^ daily). Surgical debridement was contraindicated due to profound thrombocytopenia (<10 × 10^9^/L) and coagulopathy.

Dexamethasone (10 mg/m^2^/day) was initiated for HLH but discontinued after 5 days due to accelerated fungal progression.

## 5 Follow-up and outcomes

**Figure 1:**
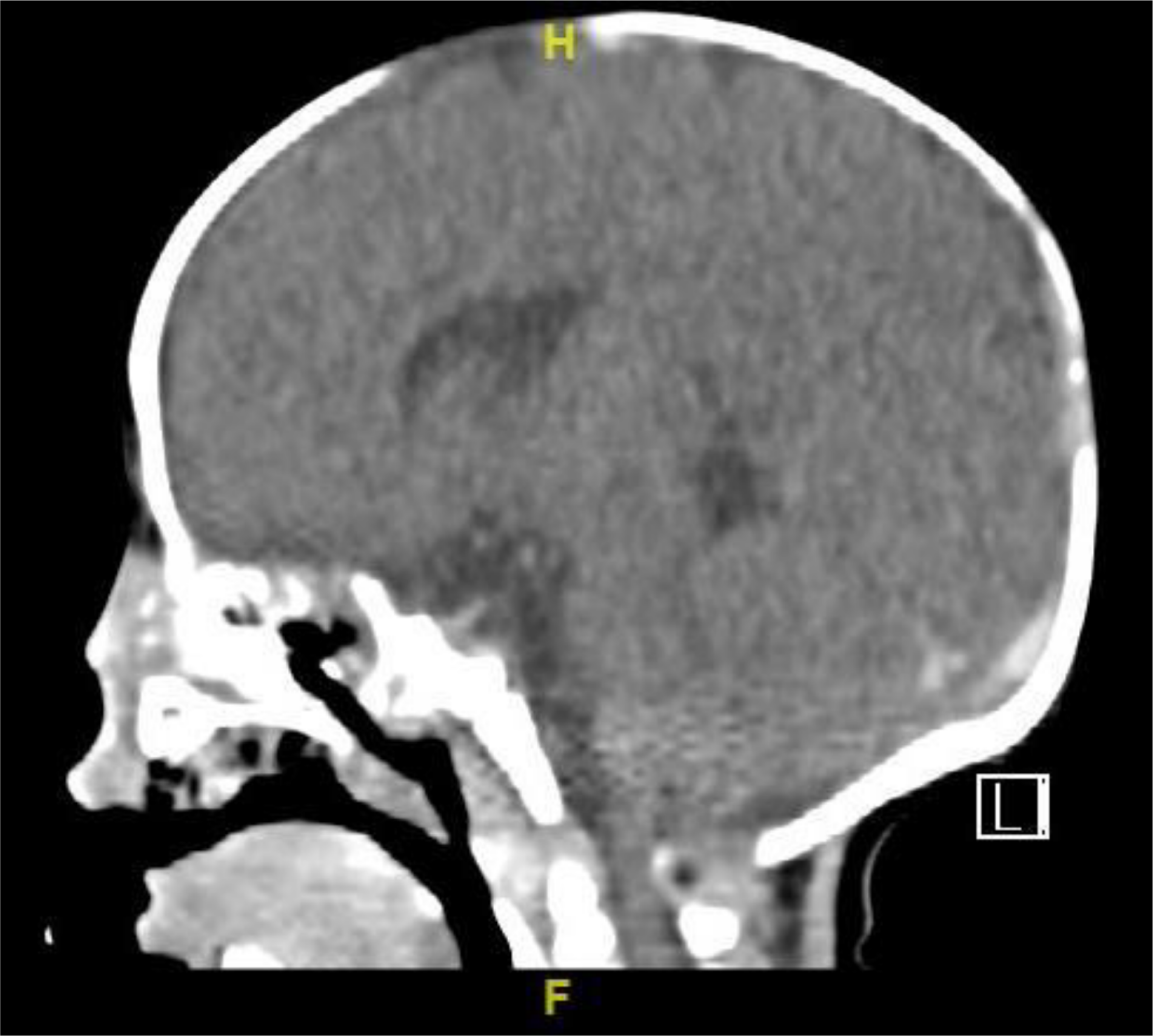
CT imaging (Day 45) demonstrating advanced invasive Aspergillus flavus sinusitis with complete craniofacial destruction involving nasal bones, hard palate, and bilateral maxillary structures.

Serial CT imaging demonstrated progressive invasive fungal sinusitis with complete destruction of nasal bones, hard palate, and bilateral maxillary structures (**Figure 1**). An HLA-identical sibling donor was identified; however, HSCT was deemed unfeasible due to uncontrolled infection, multi-organ dysfunction, and hemodynamic instability.

Following extensive family discussions, the patient was transitioned to comfort care and died at 5 months of age, 4 months after initial presentation.

## 6 Discussion

### 6.1 Strengths and limitations

Strengths: This case provides comprehensive clinical, laboratory, and genetic documentation of a novel AK2 variant with a detailed timeline following CARE guidelines. The combination of RD, HLH-like syndrome, and invasive fungal infection has not been previously reported. This case represents the first reported combination of a novel AK2 missense variant, HLH-like syndrome, and invasive fungal infection in neonatal RD, thereby expanding both the molecular and clinical spectrum of AK2 deficiency.

Limitations: Functional studies (AK2 enzyme activity, cellular ATP levels, mitochondrial membrane potential assays) were not performed due to the patient’s critical clinical condition and limited sample availability. While multiple lines of evidence including in silico predictions, complete evolutionary conservation at this position, and the severe clinical phenotype support pathogenicity, definitive mechanistic conclusions regarding inflammasome activation cannot be drawn without experimental data. Audiologic assessment was not completed due to the patient’s critical condition. HLH diagnosis relied on clinical-laboratory criteria without definitive bone marrow hemophagocytosis, potentially reflecting RD’s unique hypocellularity.

### 6.2 Comparison with medical literature

The p.Gly27Arg variant is novel. Most reported AK2 mutations are nonsense or frameshift variants causing absent protein expression (2, 12). Missense variants in catalytic domains are rare but associate with severe phenotypes (13). The affected glycine resides in the Walker A motif critical for ATP/ADP binding, and substitution with bulky arginine would disrupt nucleotide-binding geometry.

Only one prior case described RD with HLH features-a 2.5-month-old with a nonsense AK2 variant and hemophagocytosis without fungal infection (11). Our patient met 5/8 HLH-2004 criteria despite absent definitive hemophagocytosis, likely explained by profound bone marrow hypocellularity limiting target cells for phagocytosis (11, 14). The atypical histiocyte-like cells suggest aberrant mononuclear phagocyte activation.

Our findings should be considered in the context of other SCID subtypes associated with HLH. Bode et al. (15) systematically described HLH occurring in patients with various forms of SCID, including those caused by mutations in IL2RG, RAG1, IL7RA, ADA, and CD3ϵ. In most of these cases, residual T-cell or NK-cell activity was present, providing a cellular substrate for the hyperinflammatory response characteristic of HLH. What distinguishes our case and the previously reported AK2-associated RD case (11) is the occurrence of HLH-like features in the near-complete absence of both T cells. This raises the question of whether HLH-like inflammatory dysregulation in RD is driven predominantly by monocyte/macrophage-intrinsic mechanisms rather than through the classical T-cell and NK-cell cytotoxic pathway. This broadens the pathophysiological spectrum of HLH and suggests that innate immune-driven HLH may represent an underrecognized feature of severe SCID subtypes with combined innate and adaptive immune defects.

Several mechanisms may potentially explain HLH-like features in RD, though these remain speculative in the absence of functional validation. AK2 deficiency could theoretically affect mature immune cell function; mitochondrial dysfunction in macrophages has been shown in other contexts to lead to aberrant inflammasome activation (16, 17). However, whether this mechanism operates in AK2-deficient cells requires experimental confirmation. Additionally, the combination of profound immunodeficiency and uncontrolled pathogen replication may trigger excessive inflammation through toll-like receptor and inflammasome pathways (18).

Invasive fungal sinusitis in neonates is exceedingly rare, occurring almost exclusively in severe immunodeficiency (19). While Aspergillus fumigatus predominates, A. flavus causes more aggressive disease with higher mortality (20). Optimal management requires surgical debridement plus antifungals (21), but surgery was contraindicated in our patient.

### 6.3 Take-away lessons

1. RD involves complex immune dysregulation beyond simple immunodeficiency, potentially including inflammatory dysregulation manifesting as HLH-like syndrome. Notably, HLH-like features can occur in the near-complete absence of T cells and NK cells, suggesting a monocyte/macrophage-driven mechanism.
2. Clinicians should maintain high suspicion for RD in neonates with profound agranulocytosis and lymphopenia, particularly in consanguineous families.
3. Early genetic diagnosis and prompt HSCT before infectious complications are critical, as active infections preclude curative therapy.
4. Expanded newborn screening incorporating AK2 sequencing may benefit high-risk populations with consanguinity (22, 23).

## 7 Patient perspective

The patient’s parents provided the following statement:

“When our daughter was born, we had no idea anything was wrong. Within days, she became very sick. The doctors worked hard to find out what was happening, but by the time we had answers, it was too late for the transplant that could have saved her. We hope that sharing her story will help other families get diagnosed faster, so their children might have a chance at the treatment our daughter never received. We want doctors to know about this disease so no other family has to go through what we did.”

## Data Availability

The original contributions presented in the study are included in the article/supplementary material. Further inquiries can be directed to the corresponding author.
